# The moose throat bot fly *Cephenemyia ulrichii *larvae (Diptera: Oestridae) found developing in roe deer (*Capreolus capreolus*) for the first time

**DOI:** 10.1186/1751-0147-50-14

**Published:** 2008-06-02

**Authors:** Arne C Nilssen, Marja Isomursu, Antti Oksanen

**Affiliations:** 1 Tromsø University Museum, NO-9037 Tromsø, Norway; 2 Finnish Food Safety Authority Evira, Fish and Wildlife Health Research Unit (FINPAR), P.O. Box 517, FIN-90101 Oulu, Finland

## Abstract

About fifty larvae of *Cephenemyia ulrichii *Brauer (Diptera: Oestridae), some of them nearly full-grown third instars, were found in the throat of a roe deer (*Capreolus capreolus*) in June 2007 near Helsinki in Finland. The parasite is considered to be host specific, occurring only in the moose (*Alces alces*), and this paper is apparently the first report of a successful infestation in an aberrant host.

## Background

Larvae of throat, or nose or pharyngeal, bot flies in the genus *Cephenemyia *(Diptera: Oestridae) are obligate parasites that inhabit the nasal cavity, pharynx and throat of cervids [1]. In Europe there are four known species: *Cephenemyia trompe *(Modeer) in reindeer *Rangifer tarandus*; *Cephenemyia stimulator *(Clark) in roe deer *Capreolus capreolus*; *Cephenemyia auribarbis *(Meigen) in red deer *Cervus elaphus*; and *Cephenemyia ulrichii *Brauer in moose or European elk *Alces alces *[1-4]. In Finland, Sweden and Norway only *C. trompe *and *C. ulrichii *have been reported [3], with the result that *Cervus elaphus *and *Capreolus capreolus *are generally not infested by throat bot flies in Fennoscandia (see also [5]). Even though the roe deer population has increased in Finland in the recent decades [6], throat bots have never been reported in roe deer in Fennoscandia.

The moose throat bot fly *C. ulrichii *was first reported in Finland in 1910 and 1913 [7], and has subsequently become increasingly common in southern Finland [8,9]. In Sweden, *C. ulrichii *was first reported in 1988 [10], but has since increased its distribution [11]. In 1987, the species was reported for the first time in Norway (first instars in Pasvik in northeastern Norway) [3,4]. We here report a finding of moose throat bot fly larvae in roe deer.

## Methods

About 50 throat bot fly larvae were seen in an adult roe deer buck shot on 4 June 2007, in Kirkkonummi near Helsinki by the southern coast of Finland. The population consisted of different sized larvae, and three of the bigger ones were collected for laboratory examination. The other larvae were not collected.

Before we could do a proper investigation of the larvae, we considered three species possible: 1) *C. stimulator *(of roe deer), 2) *C. trompe *(of reindeer), and 3) *C. ulrichii *(of moose). As mentioned, *C. stimulator *has never been found in Finland, whereas *C. trompe *is common in the northern Finland where there are domestic reindeer, and in the east and central Finland where the wild forest reindeer (*Rangifer tarandus fennicus*) has a restricted distribution. *Cephenemyia ulrichii*, on the other hand, is seemingly abundant in the moose population (at least up to 66°N), including the Helsinki area.

The specimens, preserved in 70% ethanol, were investigated using existing keys and descriptions [1,2].

## Results and discussion

**Figure 1:**
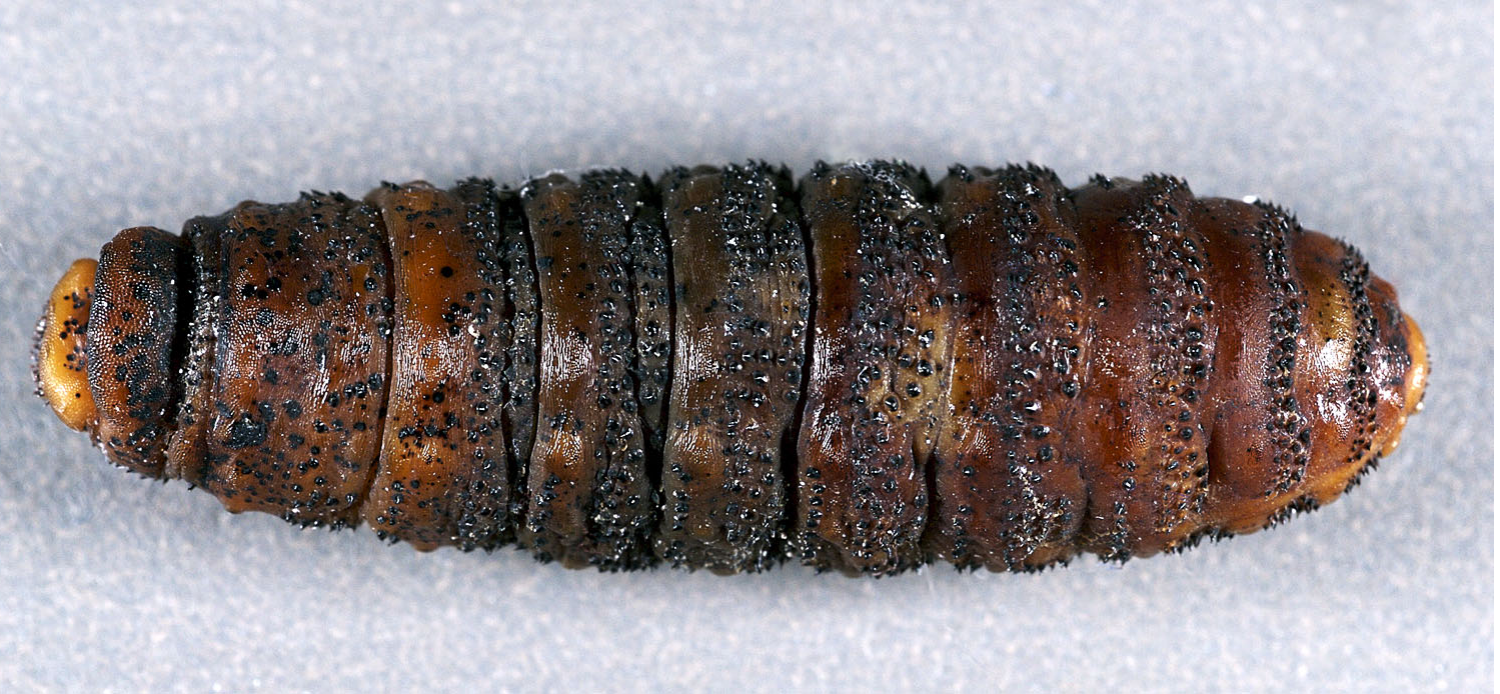
*Cephenemyia ulrichii *third instar larvae from roe deer *Capreolus capreolus*, dorsal view. Note irregular rows of spines on the anterior dorsal side. Anterior end is the thicker one.

**Figure 2:**
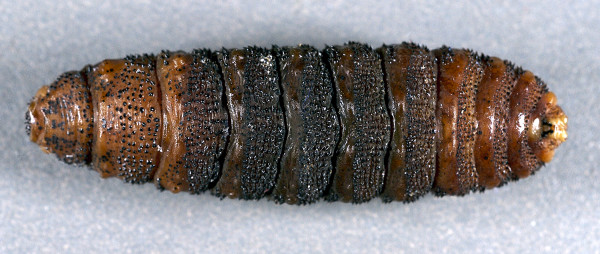
*Cephenemyia ulrichii *third instar larvae from roe deer *Capreolus capreolus*, ventral view. Note the more regular rows on the anterior ventral side.

The three larvae were about 26–27 mm long and 7 mm wide, and therefore definitively in their third and last instar. According to descriptions published [1,2], the third instar larvae of *C. ulrichii *is distinguished from *C. stimulator *and *C. trompe *by having spines irregularly placed on the anterior dorsal side. The spines on *C. stimulator *and *C. trompe *are placed in regular rows similar to those on the ventral surfaces. The posterior peritremes were also distinctive. All the larval characters coincided with the description of *C. ulrichii *(Figures 1 and 2).

Generally, all *Cephenemyia *species are very host specific and thereby also well adapted to their hosts. This host specificity is a product of the fine balance between parasite and host, and the adaptations made by the parasite to survive in the host [12]. In most cases, when an unusual host has been successfully attacked by an oestrid, the parasite will normally not develop properly. This, however, depends in part of how closely the aberrant host is related to the usual host. In the present case, roe deer and moose belong to the same family (Cervidae), although they differ considerably in size. Third instars of *C. ulrichii *reach a length of 40 mm, whereas the specific throat bot fly larvae of roe deer, *C. stimulator*, reaches a length of 30 mm [1,2], probably reflecting a restriction imposed by the size of their host.

The larvae in the present study seemed to be nearly fully grown and were probably ready to exit the host for pupation in a few weeks. The date of the find, 4 June, is probably in the middle of the exit period of the larvae. We therefore suggest that these larvae would have succeeded in developing in this aberrant host. The smaller size may either be because the larvae were not fully grown, or that the unusual and smaller host prevented the larvae from growing larger. If *C. ulrichii *larvae in roe deer are not able to reach full size, we could expect failure in pupation process or high mortality/deformation in the adult stage. It has also been observed that if larvae are taken from the host before they are mature, they often fail to pupate [13].

In the Oestridae, some species have only one host species, whereas others parasitize two or more (often closely related) host species [14]. There are several cases reported of other oestrids with seemingly successful development in unusual hosts. The reindeer warble fly, *Hypoderma tarandi*, has been found to develop into mature larvae in red deer [15], in moose [15,16], and in musk ox (*Ovibos moschatus*) [17,18]. *C. ulrichii *is regarded as very host specific for the moose [1,2], and development in aberrant hosts has apparently never been reported before. The only published reports we can find where *C. ulrichii *has attacked other species than moose, are a case in which first instar larvae were found in the conjunctival sac in the eye of a human [19], and another case where 39 young larvae were deposited by a female *C. ulrichii *on the upper lip of a human [1]. In continental Europe, several large surveys on the throat bot flies on roe deer have been performed (e.g. [20]), but only *C. stimulator *has been reported. Reindeer throat bot fly larvae have been found in the nasal cavities of dogs in Sweden [21].

Further, a middle-aged woman with ophthalmomyiasis described that while picking forest berries in eastern Finland, she got a big fly in her eye, which was subsequently affected. Following removal, the parasites were morphologically identified as 1^st ^instars of probably *C. ulrichii *(Sakari Jokiranta and Sauli Laaksonen, unpublished).

## Conclusion

These unusual oestrid – host relationships in Cervidae and other animals, including humans (see review [22]), may be more common than so far considered because, in the normal hunting season in the autumn, the larvae are very small and difficult to detect without specific methods. As a result, even experienced hunters would be unaware of the existence of oestrid fly larvae even in their primary host species. Most reported aberrant host cases, however, only inform that unusual host can be infected, whereas successful development, as the present case, is rarely reported.

## Competing interests

The authors declare that they have no competing interests.

## Authors' contributions

AN was responsible for the parasitological examination and identification of the Oestrid fly larvae, MI handled the hunter-laboratory interface. All authors were involved in drafting the manuscript and gave final approval of the manuscript.
